# Establishment of a protocol to administer immunosuppressive drugs for iPS cell-derived cardiomyocyte patch transplantation in a rat myocardial infarction model

**DOI:** 10.1038/s41598-023-37235-5

**Published:** 2023-06-29

**Authors:** Emiko Ito, Ai Kawamura, Takuji Kawamura, Maki Takeda, Akima Harada, Noriko Mochizuki-Oda, Yoshiki Sawa, Shigeru Miyagawa

**Affiliations:** Department of Cardiovascular Surgery, Osaka University Graduate School of Medicine, 2-2 Yamadaoka, Suita, Osaka 565-0871 Japan

**Keywords:** Stem cells, Transplant immunology

## Abstract

Transplantation of human allogeneic induced pluripotent stem cell-derived cardiomyocytes (hiPSC-CMs) is a new, promising treatment for severe heart failure. However, immunorejection is a significant concern in allogeneic hiPSC-CM transplantation, requiring the administration of several immunosuppressive agents. An appropriate protocol for the administration of immunosuppressants may substantially affect the efficacy of hiPSC-CM transplantation in case of heart failure owing to allogeneic transplantation. In this study, we investigated the effect of immunosuppressant administration duration on the efficacy and safety of allogenic hiPSC-CM patch transplantation. We used a rat model of myocardial infarction to evaluate cardiac function using echocardiography six months after the transplantation of hiPSC-CM patches with immunosuppressant administration for either two or four months and compared them to control rats (sham operation, no immunosuppressant administration). Histological analysis performed at 6 months after hiPSC-CM patch transplantation revealed significant improvement in cardiac function in immunosuppressant-treated rats compared with those in the control group. Moreover, fibrosis and cardiomyocyte size was significantly reduced and the number of structurally mature blood vessels was significantly increased in the immunosuppressant-treated rats compared to control rats. However, there were no significant differences between the two immunosuppressant-treated groups. Our results show that prolonged administration of immunosuppressive agents did not enhance the effectiveness of hiPSC-CM patch transplantation, and therefore, highlight the importance of an appropriate immunological regimen for the clinical application of such transplantation.

## Introduction

Despite significant progress in medical sciences, heart failure remains the leading cause of death worldwide. Myocardial regeneration therapy has been attracting attention for approximately 20 years and is expected to be used as a novel treatment for severe heart failure, which can prolong patient survival and improve their quality of life^1,2^. In recent years, cell transplantation has been reported to be useful for restoring cardiac function in patients with severe heart failure, and clinical applications of auto-skeletal myoblasts have already been initiated^3–5^. In addition, embryonic stem cells and induced pluripotent stem cells are expected to be used in regenerative medicine because they possess the pluripotency to differentiate into many types of cells that comprise the body^6–8^.

Human induced pluripotent stem cells (hiPSCs), developed in 2007, are considered a promising tool that can supply the cells lost in diseased organs^9–11^. Although autologous hiPSCs have a low rate of rejection, it is time-consuming and costly to confirm the quality and safety of hiPSC-derived products. Due to this issue, allogeneic hiPSC-derived products may be more clinically suitable than autologous hiPSCs^12,13^. Although Human Leukocyte Antigen haplotype-homozygous (HLA-homo) allogeneic hiPSCs have been developed to reduce immune rejection, cardiac myocytes derived from HLA-homo hiPSCs were reportedly rejected after in vivo transplantation^14,15^. Therefore, immunosuppressive agents and therapies are critical for the clinical application of allogeneic hiPSCs in regenerative medicine.

The administration of immunosuppressants is necessary for the transplantation of allogeneic hiPSCs as well as for heart transplantation. The basic immunotherapy for heart transplantation is a triple therapy consisting of cyclosporine or tacrolimus (TAC), mycophenolate mofetil (MMF), and prednisolone (PSL)^16^. However, the appropriate immunologic regimens and duration of immunosuppressive medication for therapies using allogeneic hiPSCs have not been fully elucidated. In a primate heart failure model, an HLA-matched transplant of HLA-homo monkey iPSC-derived cardiomyocyte sheets was rejected within four months despite administration of immunosuppressive agents, however, improvement in cardiac function was maintained^15^. As described above, excessive administration of immunosuppressive drugs, which has potential side effects, such as infection and malignancies, is conceivably not required over a long period to achieve clinical success using allogeneic hiPSCs for improving cardiac function and safety. Therefore, optimizing the protocol for the administration of immunosuppressive drugs is needed to ensure safety and efficacy for the clinical application of human induced pluripotent stem cell-derived cardiomyocytes (hiPSC-CMs).

In this study, we investigated the effect of the duration of administration of immunosuppressive agents for safe and effective hiPSC-CM patch transplantation to develop an appropriate immunological protocol for the clinical application of allogeneic hiPSC cell-derived cardiac products.

## Results

### Characterization of human iPSC-derived cardiomyocytes

**Figure 1:**
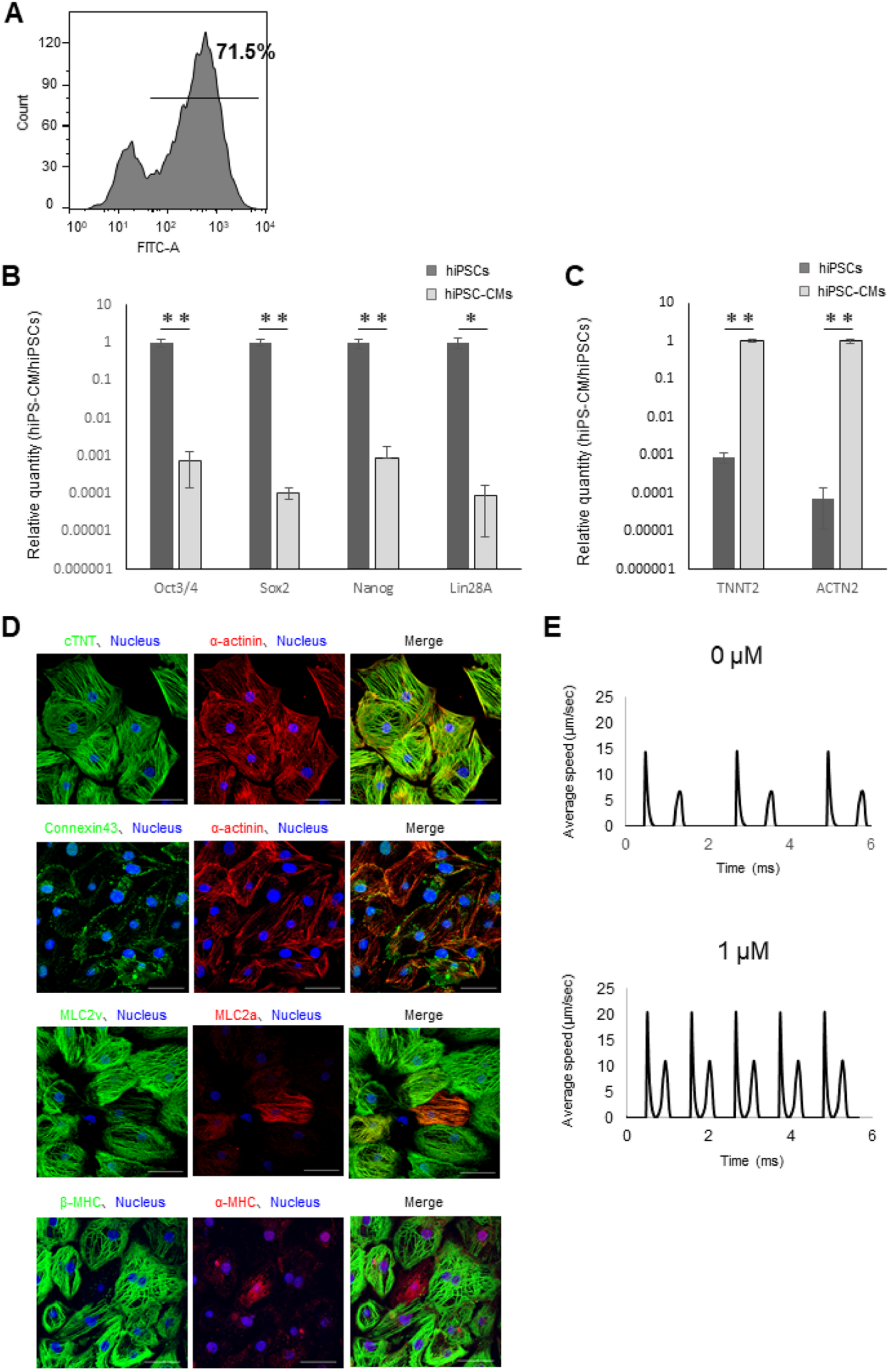
In vitro characterization of human induced pluripotent stem cell (iPSC)-derived cardiomyocytes. (**A**) Representative flow cytometry data for hiPSC-CMs labeled with anti-cTnT antibody. (**B**,**C**) Comparisons of gene expression in iPS and iPS-CM using RT-qPCR for (**B**) markers of pluripotency, Oct3/4, SOX2, NANOG, and Lin28A and (**C**) markers of cardiomyocytes, TNNT2 and ACTN2. Data are from one representative experiment for each marker and are normalized to peak expression ± SD. *P < 0.05, **P < 0.01, using unpaired Student's *t*-test. (**D**) The structure and morphology of iPS-CMs. Immunolabeling of hiPSC-CMs shows expression of cardiac-specific proteins, cardiac troponin T (green) and α-actinin (red), connexin-43 (green), and α-actinin (red), MLC2V (green) and MLC2A (red), and β-MHC (green) and α-MHC (red) along with Hoechst 33342 staining. Scale bar 50 μm. (**E**) Contraction properties of hiPSC-CMs upon addition of drugs. Representative contraction waveform after the addition of isoproterenol.

Differentiation of hiPSCs into cardiomyocytes was induced using several recombinant proteins and compounds. A total of 71.5% cardiomyocytes were positive for the cardiac marker, cardiac troponin T (cTnT), as determined by fluorescence-activated cell sorting (FACS) (Fig. 1A). The expression of the stem cell markers octamer-binding protein (Oct)3/4, sex-determining region-box (Sox)2, NANOG, and Lin-28 Homolog A (Lin28A), decreased with the progression of cardiac differentiation as determined via RT-qPCR (Fig. 1B). On the contrary, the expression of the cardiomyocyte markers, troponin T2, cardiac type (TNNT2), and actinin alpha 2 (ACTN2), increased with the progression of cardiac differentiation (Fig. 1C). In addition, immunohistochemical staining revealed that the hiPSC-CMs expressed cardiac-specific structural proteins, such as cTnT, α-actinin, MLC2a, MLC2v, α-MHC, β-MHC, and the gap junction protein, connexin 43 (Fig. 1D). The cell motion imaging system revealed that the change in contractile properties of hiPSC-CMs was significantly increased upon addition of 1 μM isoproterenol (Fig. 1E).

### Cardiac function after hiPSC-CM patch transplantation to a rat MI model

**Figure 2:**
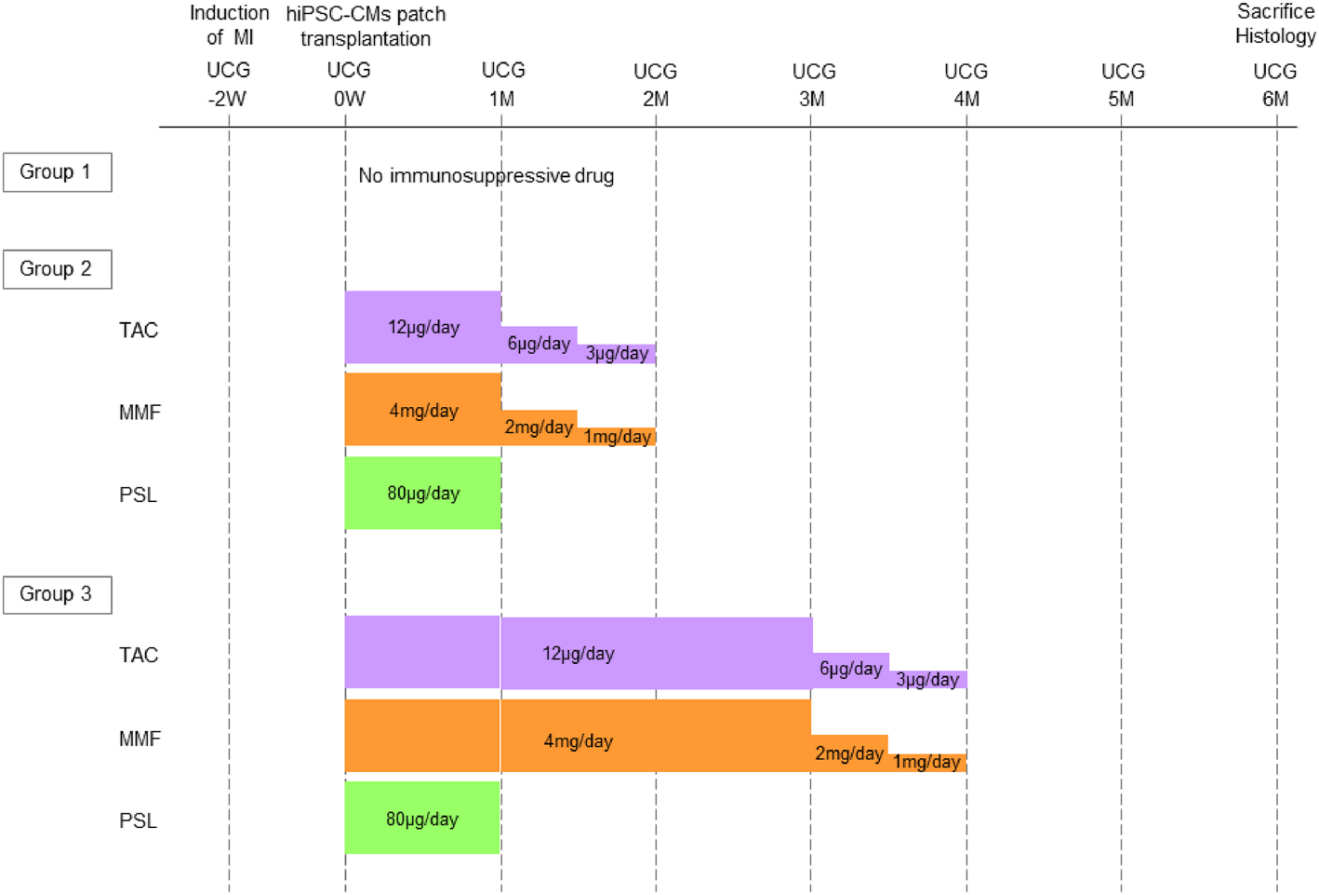
Study protocol and immunosuppressive drug administration regimen, evaluation of cardiac function, and histological analysis in vivo. Transplantation of hiPSC-CM patches on the surface of the left ventricle (LV) in an ischemic cardiomyopathy rat model. After MI induction, rats were divided into three groups as follows: no immunosuppressants (Group 1); treated with tacrolimus (TAC), mycophenolate mofetil (MMF), and prednisolone (PSL) for 1 month and 1-month tapering period (Group 2); and treated with TAC and MMF for 3 months, PSL for 1 month and 1-month tapering period (Group 3).

We implanted hiPSC-CM patches on the infarct and border areas of the heart. Cardiac function was evaluated using echocardiography before surgery (at the baseline), two weeks after left coronary artery ligation, and 1, 2, 3, 4, 5, and 6 months after hiPSC-CM patch transplantation or sham operation (Fig. 2).

**Figure 3:**
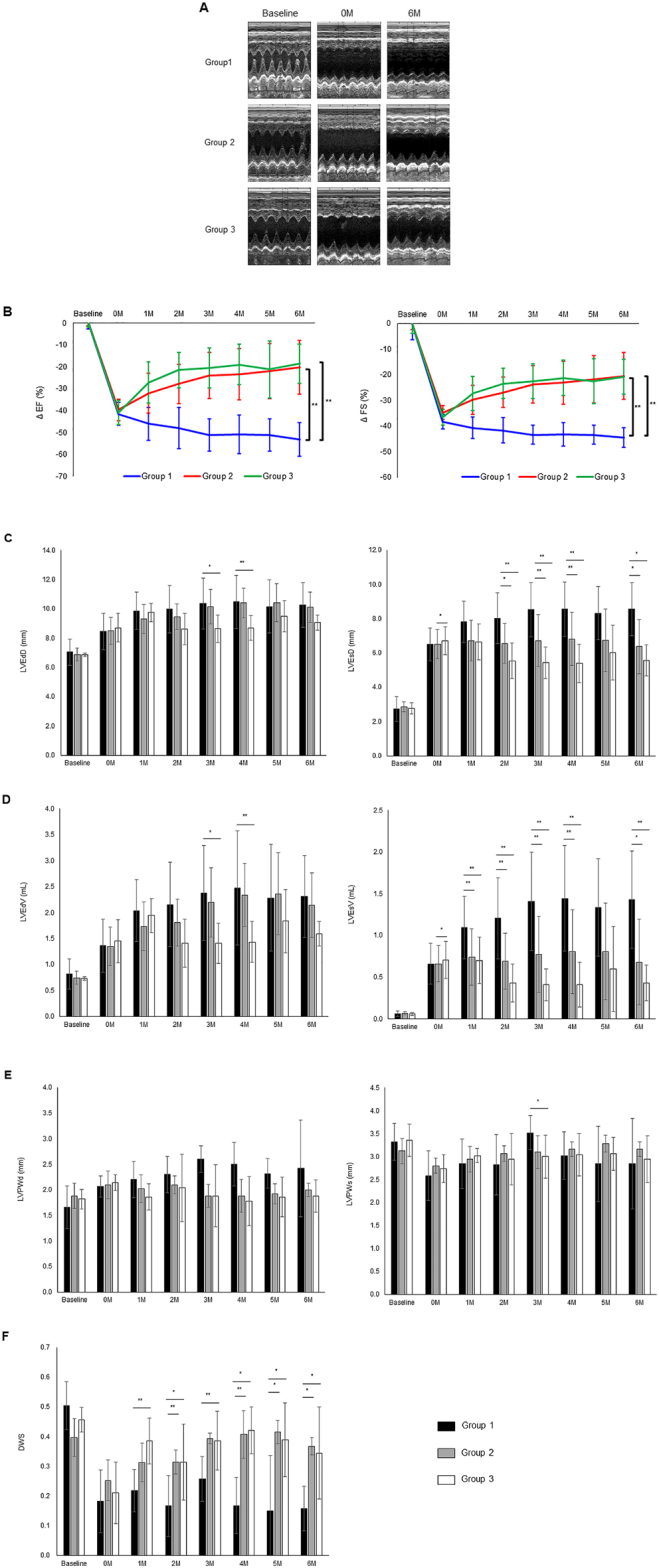
Evaluation of cardiac function following hiPSC-CM patch transplantation using echocardiography. (**A**) Representative M-mode images at the baseline (before induction of MI), 0 M (14 days after LAD coronary artery ligation and before hiPSC-CM patch transplantation), and 6 M (6 months after hiPSC-CM patch transplantation) for each group. (**B**) Quantification of the rate of change from the baseline of left ventricular ejection fraction (LVEF) and fractional shortening (FS). Quantification of left ventricular end-systolic diameter (LVESD) and left ventricular end-diastolic diameter (LVEDD) (**C**), left ventricular end-systolic volume (LVESV), and left ventricular end-diastolic volume (LVEdV) (**D**), left ventricular posterior wall end-systole (LVPWs), and, left ventricular posterior wall end diastole (LVPWd) (**E**), and diastolic wall strain (DWS) (**F**). Data are normalized to peak expression ± SD. *P < 0.05, **P < 0.01.

The cardiac function at the baseline and after the induction of MI did not differ significantly among the three groups. In Group 1, LVEF, FS, LVESD, LVEDD, LVESV, LVEDV, LVPWs, LVPWd, and DWS deteriorated in a time-dependent manner. Whereas, LVEF and FS were significantly increased one month after hiPSC-CM patch transplantation in Groups 2 and 3. Significant improvement was observed in Group 2 (changes in LVEF (ΔLVEF) and FS (ΔFS) at 1 month and after MI: ΔLVEF; 8.4% ± 5.1% [P < 0.01] and ΔFS; 5.6% ± 3.6% [P < 0.05]) and Group 3 (ΔLVEF; 11.6% ± 11.2% [P < 0.01] and ΔFS; 8.3% ± 8.3% [P < 0.01]) compared to Group 1 (ΔLVEF; − 3.8% ± 4.1% and ΔFS; − 2.0% ± 2.2%) (Fig. 3A,B). There were no significant differences between the rate of change in Group 2 and Group 3 (ΔLVEF; P = 0.66, ΔFS; P = 0.55). The improved LVEF and FS values were sustained for at least 6 months in Groups 2 and 3. Six months after hiPSC-CM patch transplantation, the LVESD and LVESV were significantly lower in Group 2 (changes in LVESD (ΔLVESD) and LVEDV (ΔLVESV) at 6 months versus after MI: ΔLVESD; 3.5 ± 1.7 mm [P < 0.05] and ΔLVESV; 0.6 ± 0.5 mL [P < 0.05]) and Group 3 (ΔLVESD; 2.8 ± 1.2 mm [P < 0.05] and ΔLVESV; 0.4 ± 0.2 mL [P < 0.01]) compared to Group 1 (ΔLVESD; 5.8 ± 1.7 mm and 1.4 ± 0.6 mL) (Fig. 3C,D). However, there were no significant differences between Group 2 and Group 3 (ΔLVESD; P = 0.93, ΔLVESV; P = 0.46). The LVEDD, LVEDV, LVPWs, and LVPWd were not significantly different among the three groups (Fig. 3C–E). Six months after hiPSC-CM patch transplantation, DWS was significantly lower in Group 2 (0.37 ± 0.03 [P < 0.05]) and Group 3 (0.34 ± 0.03 [P < 0.05]) than in Group 1 (0.16 ± 0.15). However, there were no significant differences between Group 2 and Group 3 (P = 0.94) (Fig. 3F).

### Evaluation of cardiac fibrosis and transverse diameter of cardiac myocytes in the infarcted area

**Figure 4:**
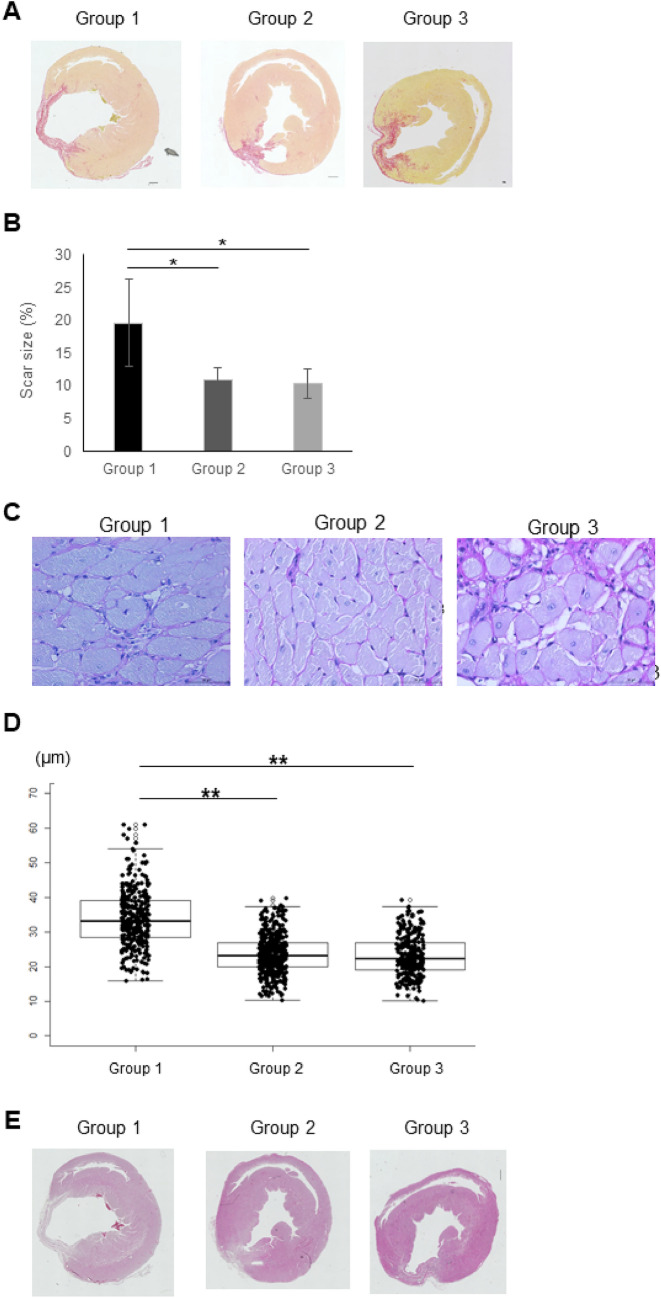
Histological evaluation of left ventricular peri-infarct myocardium 6 months after hiPSC-CM patch implantation. (**A**,**B**) Fibrosis was evaluated by Masson’s trichrome staining. Representative image of Masson's trichrome staining (**A**) and the quantification of ratio of fibrotic area to the whole heart area (**B**). Scale bar 100 μm. *P < 0.05, **P < 0.01. (**C**,**D**) Diameter of the cardiomyocytes was evaluated by Periodic acid-Schiff (PAS) staining. Representative image of PAS staining (**C**) and the quantification of cardiomyocyte diameter (**D**). Scale bar 100 μm. *P < 0.05, **P < 0.01. (**E**) Tumorigenicity was evaluated by hematoxylin and eosin (H&E) staining. Representative image of H&E staining.

Fibrosis in extracted rat heart tissue 6 months after hiPSC-CM patch transplantation was assessed using Masson's trichrome staining (Fig. 4A). The percentage of fibrosis was significantly reduced in Group 2 (11.0% ± 1.8% [P < 0.05]) and Group 3 (10.4% ± 2.2% [P < 0.05]) compared to that in Group 1 (19.6% ± 6.7%) (Fig. 4B). However, there was no significant difference in the reduction of fibrosis between Group 2 and Group 3 (P = 0.9). The transverse diameter of cardiac myocytes in the border region of myocardial infarct was evaluated by PAS staining (Fig. 4C). The diameter of the cardiomyocytes was significantly reduced in Group 2 (22.8 μm [IQR, 21.0–24.6 μm] (P < 0.01)) and Group 3 (23.3 μm [IQR, 21.5–25.1 μm] (P < 0.01)) compared with that in Group 1 (35.3 μm [IQR, 31.8–38.8 μm]) (Fig. 4D). There were no significant differences between Group 2 and Group 3 (P = 0.9). The formation of teratoma within the infarcted area after hiPSC-CM patch transplantation was assessed via H&E staining, which revealed no tumors 6 months after hiPSC-CM patch transplantation in Groups 2 and 3 (Fig. 4E).

### Angiogenesis and arteriogenesis in peri-infarct myocardium

**Figure 5:**
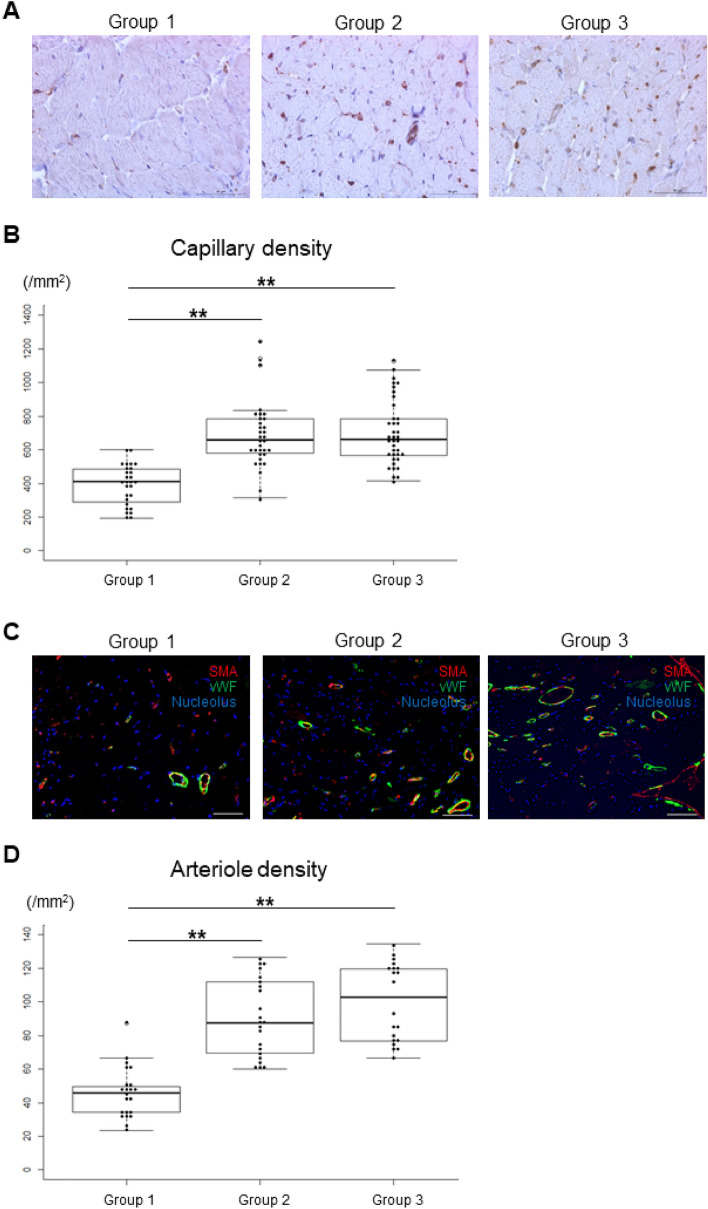
Evaluation of angiogenesis and arteriogenesis of the left ventricle in the peri-infarct myocardium 6 months after hiPSC-CM patch implantation. (**A**,**B**) Capillaries immunostained with anti-von Willebrand Factor (vWF) antibody to assess angiogenesis. Representative image of vWF staining (brown) (**A**) and the quantification of the number of vWF-positive cells (**B**). Scale bar 1000 μm. *P < 0.05, **P < 0.01. (**C**,**D**) Mature vessel immunostained with anti-vWF and α-smooth muscle actin (SMA) antibodies to assess arteriogenesis. Representative image of vWF (green) and αSMA (red) staining (**C**) and the quantification of the number of vWF and αSMA double-positive cells (**D**). Scale bar 100 μm. *P < 0.05, **P < 0.01.

MI causes a significant loss of vascularity. We investigated the mechanism underlying the beneficial effects of hiPSC-CM patch transplantation at the site of MI by analyzing angiogenesis and arteriogenesis in peri-infarct myocardium 6 months after the transplantation of hiPSC-CM patches. The capillary density, as detected by the number of vWF-positive vascular structures/mm^2^ in the border region, was significantly higher in Group 2 (684.8 [IQR, 487.1–882.5] (P < 0.01)) and Group 3 (700.0 [IQR, 508.5–891.5] (P < 0.01)) than in Group 1 (392.8 [IQR, 265.0–500.6]) (Fig. 5A,B). There were no significant differences in the number of vWF-positive vascular structures between the immunosuppressant-treated groups. The arteriole density, as detected by the number of vWF/SMA double-positive vessels/mm^2^ in the border region, was significantly higher in Group 2 (90.4 [IQR, 67.7–113.1] (P < 0.01)) and Group 3 (100.1 [76.9–123.3] (P < 0.01)) than in Group 1 (45.9 [IQR, 31.0–60.8]) (Fig. 5C,D). There were no significant differences in the number of vWF/SMA double-positive vessels between the immunosuppressant-treated groups. These data suggest that iPSC-CM patch transplantation induced neovascularization and arteriolar capillary formation in the border region of MI, leading to suppression of cardiomyocyte hypertrophy and remodeling after MI.

## Discussion

In this study, cardiac myocytes derived from hiPSCs were transplanted to an ischemic cardiomyopathy rat model with normal immune function to investigate the appropriate duration of immunosuppressive medications for clinical application. We did not observe differences in cardiac function, inhibition of fibrosis, or increase in capillary density after the two different durations of immunosuppressive treatment. The cardiac function was maintained for at least 6 months, and this functional recovery was not correlated with the duration of immunosuppressive medication.

Transplantation of cardiac myocytes derived from hiPSCs was expected to have a considerable effect on the direct contribution to the improvement of contractile function by electrical integration between transplanted grafts and recipient myocardium in addition to cytokine-induced angiogenesis in severe heart failure with few cardiac myocytes^19–21^. We used PSL, TAC, and MMF as the immunosuppressant regimen for heart transplantation and expected to observe the long-term survival of the transplanted myocardial tissue. However, long-term engraftment of myocardial tissue was not observed after the two different durations of immunosuppressant administration. Since there was no difference in cardiac function recovery between two different durations of immunosuppressant administration, we speculate that the transplanted patch disappeared within 2 months. Our results suggest that the duration of administration of immunosuppressants does not correlate with long-term engraftment of myocardial tissue, indicating that the improvement in cardiac function in immunosuppressed xenotransplant might largely depend on angiogenesis caused by cytokines.

In ischemic heart disease, angiogenesis induced by various cytokines including hepatocyte growth factor, vascular endothelial growth factor, fibroblast growth factor, and stromal cell-derived factor 1, secreted by transplanted cells, has been reported to be the major mechanism for myocardial regeneration in various cell therapies^22–24^. Angiogenesis in the damaged myocardium that receives cell sheets may improve cardiac function by increasing the blood supply to the ischemic myocardium and activating cardiac myocytes in a hibernating state. In the present study, no differences in cardiac function, antifibrotic activity, capillary density, and the number of mature blood vessels were observed for different durations of immunosuppressive medication 6 months after transplantation. Thus, most angiogenesis by paracrine effects of cytokines could occur early after transplantation, thus increasing the number of mature blood vessels after transplantation, and this mature vascular system persists until the endpoint, which may enable achieving cardiac functional recovery despite the disappearance of transplanted cells.

In a cynomolgus monkey model of MI, major histocompatibility complex (MHC)-matched cynomolgus macaque iPSC-CMs demonstrated prolonged graft survival upon immunosuppressant administration compared with no immunosuppressant administration^14,15^. This observation indicates that the use of immunosuppressive agents may be essential, even when the donor and recipient are MHC-matched. In addition, cardiomyocyte sheet-derived MHC-matched cynomolgus macaque iPSCs led to an equal improvement in cardiac function compared with MHC-mismatched cardiomyocyte sheets under immunosuppressive medication in a cynomolgus monkey MI model^15^, suggesting that transplantation of MHC-mismatched iPSC-CMs with the administration of immunosuppressive agents has no relevance in clinical practice.

The disappearance of transplanted graft primarily depends on ischemia caused by poor angiogenesis at the implant site, inflammation associated with oxidative stress, and the release of cytotoxic cytokines^25–27^. Post-transplant graft retention requires the formation of blood vessels that perfuse the entire graft with oxygen and nutrients^24–32^. We previously reported the use of a combination of cell sheets and omentum transplantation for improving the blood flow through the graft^24–26,28^. Because the omentum is a vascularized organ that contains abundant angiogenic factors and has anti-inflammatory effects, the transplanted tissue may provide adequate blood supply, nutrition, and anti-inflammatory effects. The combined implantation of iPS myocardial cell sheets and omentum in a pig model of MI improved cell survival at the transplant site compared with iPS cardiomyocyte sheet implantation alone. Moreover, it also increased angiogenesis and expression of angiogenesis-related factors in the transplant area, resulted in arteriogenesis, and stabilized the cells in the bloodstream^27,29^.

Minimizing the use of immunosuppressants could be necessary to reduce their side effects because the degree of improvement in cardiac function is not associated with the duration of immunosuppressant administration. Unlike somatic cells, such as mesenchymal stem cells, hiPSC-CMs can induce cytokine-mediated angiogenesis as well as contribute to contractile force in heart failure by electrical and functional integration with the recipient's myocardium at the transplant site. Therefore, optimal immunosuppression, including MHC-modified hiPSC-CM transplantation and immunosuppressive drug regimen, and sufficient blood supply are crucial for the long-term survival of grafts. Further studies concerning immunology and tissue perfusion may be warranted to improve clinical outcomes after cell transplantation therapies.

## Conclusions

In conclusion, recovery of cardiac function was detected and maintained even after the disappearance of the implanted hiPSC-CM patches in rats with immunosuppressive agents. Since the prolonged administration of immunosuppressive agents did not show any additional effect on cardiac recovery, the duration of immunosuppressive agents should not be prolonged, although further examinations are warranted.

## Materials and methods

### Cell culture and cardiac differentiation of hiPSCs

We used a human iPSC line, QHJI14s04, derived from human mononuclear cells, which was provided by Professor Yamanaka, Kyoto University, Kyoto, Japan. The QHJI14s04 cells were cultured on iMatrix511 (Nippi, Tokyo, Japan)-coated dishes in Stem Fit Ak03N (Ajinomoto, Tokyo, Japan). The cells were differentiated according to previously published methods^17^. Briefly, we generated embryoid bodies (EBs) from hiPSCs in an EZ sphere (Iwaki, Shizuoka, Japan), and cultured them with various recombinant proteins and chemical compounds for cardiomyogenic differentiation in 100 mL bioreactors (Able corp., Tokyo, Japan) for 16 days. After differentiation, cardiomyocyte aggregates were purified by culturing in glucose-free Dulbecco’s modified Eagle’s medium (DMEM; Nacalai Tesque, Kyoto, Japan) for 7 days. Afterward, the cells were dissociated and the dissociated cells were cultured in DMEM supplemented with 10% fetal bovine serum (FBS; Sigma-Aldrich, St. Louis, MO) and brentuximab vedotin (ADCETRIS™, Takeda, Osaka, Japan) for 5 days to eliminate residual undifferentiated cells. The cells were suspended in a cell banker (Nippon Genetics, Tokyo, Japan) and frozen using a programmed freezer (FZ2000; STREX Inc. Osaka, Japan). After freeze-thawing, hiPSC-CMs were used for all experiments.

### Flow cytometry

The hiPSC-CMs were fixed with CytoFix fixation buffer (BD Biosciences, NJ, USA) and permeabilized with Perm/Wash buffer (BD Biosciences). The cells were subsequently stained with unconjugated mouse anti-cardiac isoform of troponin T (cTnT) (Santa Cruz Biotechnology, TX, USA) in Perm/Wash buffer. Alexa Fluor 488 goat anti-mouse antibody (Thermo Fisher Scientific, Waltham, MA, USA) was used as the secondary antibody. The cells were analyzed using a fluorescence-activated cell sorting (FACS) Canto II system (BD Biosciences). Data were analyzed using FlowJo software (Tree Star, OR, USA).

### Immunofluorescent staining

The hiPSC-CMs were fixed with 4% paraformaldehyde (Fujifilm Wako Pure Chemical Corporation, Osaka, Japan) and incubated with anti-cTnT (Abcam, Cambridge, UK), anti-sarcomeric alpha actinin (α-actinin) (Sigma-Aldrich, St. Louis, MO, USA), anti-connexin 43 (Sigma-Aldrich), anti-myosin light chain 2a (MLC2a) (Synaptic Systems GmbH**,** Goettingen**,** Germany), anti-myosin light chain 2v (MLC2v) (Proteintech Group, Rosemont, IL, USA), anti-alpha cardiac myosin heavy chain (α-MHC) (Sigma-Aldrich), or anti-beta cardiac myosin heavy chain (β-MHC) (Sigma-Aldrich) as primary antibodies, followed by secondary antibodies, namely AlexaFluor488 or AlexaFluor555 conjugated donkey anti-rabbit or anti-mouse (Thermo Fisher Scientific) antibodies. Nuclei were counterstained with Hoechst 33342 (Dojindo, Kumamoto, Japan). Images of the stained cell samples were captured using a confocal laser scanning microscope FV10i (Olympus; Tokyo, Japan).

Rat hearts transplanted with hiPSC-CM patches were fixed in 10% buffered formalin (Fujifilm) and embedded in paraffin using a Microm STP 120 Spin Tissue Processor (STP120-3; Thermo Fisher Scientific). Serial paraffin-embedded sections were cut at a thickness of 0.5 μm using a Microm HM 430 system (MIC 990010; Thermo Fisher Scientific), deparaffinized in xylene (Fujifilm), dehydrated in a graded series of ethanol (Fujifilm), and stained with hematoxylin and eosin (H&E; Muto Pure Chemicals). The sections were immersed in methanol (Fujifilm) containing 3% hydrogen peroxide (Fujifilm) and the slides were incubated overnight at 4 °C with anti-cTnT (Thermo Fisher Scientific) and anti-human specific cTnT (Abcam) as primary antibodies, followed by secondary antibodies, namely Alexa Fluor 488 or Alexa Fluor 555 conjugated donkey anti-rabbit or anti-mouse (Thermo Fisher Scientific) antibodies. Nuclei were counterstained with Hoechst 33342 (Dojindo, Kumamoto, Japan). Images of the rat hearts were captured using the BZ-X810 analyzer (Keyence; Osaka, Japan).

### RNA extraction and reverse transcription quantitative real-time polymerase chain reaction (RT-qPCR)

Total RNA was extracted from hiPSCs and hiPSC-CMs after freeze-thawing using the RNeasy Mini Kit (Qiagen, Hilden, Germany) and was reverse transcribed to cDNA using the Super Script VILO cDNA Synthesis kit (Thermo Fisher Scientific, Waltham, MA). RT-qPCR was performed using a ViiA 7 Real-Time PCR System (Applied Biosystems, Foster City, CA) using SYBR Green (Applied Biosystems). The primer sequences are listed in Table 1. Each sample was analyzed in triplicate for each of the studied genes. The average copy number of gene transcripts was determined using glyceraldehyde-3-phosphate dehydrogenase as a control for each sample.

**Table 1:** List of primers used for quantitative real-time PCR.

Gene symbol	Primers	
Human		
*GAPDH*	SYBR	F: 3′-CAATGACCCCTTCATTGACC-5′ R: 5′-TTGATTTTGGAGGGATCTCG-3′
*LIN28*	SYBR	F: 3′-CACGGTGCGGGCATCTG-5′ R: 5′-CCTTCCATGTGCAGCTTACTC-3′
*NANOG*	SYBR	F: 3′-CTCAGCTACAAACAGGTGAAGAC-5′ R: 5′-TCCCTGGTGGTAGGAAGAGTAAA-3′
*OCT4*	SYBR	F: 3′-GAAACCCACACTGCAGCAGA-5′ R: 5′-TCGCTTGCCCTTCTGGCG-3′
*SOX2*	SYBR	F: 3′-GCGCCCTGCAGTACAACTC-5′ R: 5′-CGGACTTGACCACCGAACC-3′
*TNNT2*	SYBR	F: 3′-GGCAGCTCCTGTTTGGAAATG-5′ R: 5′-TTATTACTGGTGTGGAGTGGGTGTG-3′
*ACTN2*	SYBR	F: 3′-TTTCCCTGTGTGTTGGTTGC-5′ R: 5′-TGATTACACTCCGCACATTTCA-3′

### Assessment of the contraction property of hiPSC-CMs

The hiPSC-CMs were seeded onto 96-well plates at a density of 1 × 10^5^ cells/well. Cell motion analysis was performed using a Cell Motion Imaging System (SI8000; SONY, Tokyo, Japan), as previously described^18^. Isoproterenol (Calbiochem Merck Millipore) was added to the culture medium and the hiPSC-CMs were monitored for 30 min after the addition of the drug.

### Preparation and transplantation of hiPSC-CMs patches into myocardial infarction (MI) rat model

The animal experiment was conducted in compliance with the animal experiment regulations. Prior to seeding the cells, the surface of temperature-responsive dishes (UpCell; CellSeed, Japan) was coated with FBS overnight. After freeze-thawing, hiPSC-CMs were plated onto a 12-well plate UpCell at a density of 4 × 10^6^ cells/cm^2^ in DMEM containing 20% FBS and cultured at 37 °C in an atmosphere containing 5% CO_2_. After culturing for 72 h, hiPSC-CM patches were harvested and washed gently with Hank’s balanced salt solution (Thermo Fisher Scientific, Waltham, MA, USA). Male Sprague–Dawley (SD) rats (8–10-week-old, weighing 250–350 g, Japan SLC, Shizuoka, Japan) were subjected to left thoracotomy under general anesthesia induced with a mixture of three types of inhalation anesthetics, and were intubated and placed on a respirator during surgery to maintain ventilation with oxygen and 3% isoflurane inhalation anesthesia. The left anterior descending artery of SD rats was permanently ligated through a left thoracotomy to prepare a MI model. Two weeks later, the rats were randomly divided into a sham group (Group 1) or two immunosuppressant treatment groups (Groups 2 and 3) after echo measurement (N = 10). The experimental design and protocols used in this study are shown in Fig. 2. The hiPSC-CM patch was transplanted to the left ventricular MI site under anesthesia in the two immunosuppressant treatment groups. The transplanted hiPSC-CM patches covered both the infarct and border areas of the heart and were fixed with fibrin glue. After surgery, all the rats were allowed to recover in individual temperature-controlled cages. The rats were euthanized six months later. All surgeries and euthanasia were performed under deep anesthesia to minimize animal suffering.

### Immunosuppressive drug administration regimen

Rats were divided into three groups as follows: no immunosuppressant administration (Group 1); treated with PSL for 1 month, followed by TAC and MMF for 1 month, and a 1-month tapering period (Group 2); treated with PSL for 1 month, followed by TAC and MMF for 3 months and 1-month tapering period (Group 3). Oral administration of TAC (12 μg/day), MMF (8 mg/day), and PSL (80 μg/day) was performed the day after hiPSC-CM patch transplantation. During the 1-month tapering period, TAC was gradually reduced to 6 μg/day for 2 weeks and 3 μg/day for 2 weeks, MMF was gradually reduced to 4 mg/day for 2 weeks and 2 μg/day for 2 weeks, and PSL was not administered.

### Echocardiography

Transthoracic echocardiography was performed using Nolus (Hitachi, Tokyo, Japan) under inhalation anesthesia with isoflurane, 2 weeks after coronary artery ligation (just before transplantation) and 1, 2, 3, 4, 5, and 6 months after transplantation of hiPSC-CM patches. Left ventricular end-systolic diameter (LVESD), left ventricular end-diastolic diameter (LVEDD), left ventricular end-systolic volume (LVESV), left ventricular end-diastolic volume (LVEDV), left ventricular posterior wall thickness at end-systole (LVPWs), and left ventricular end-diastolic posterior wall dimension (LVPWd) were automatically calculated using the echo device. Left ventricle ejection fraction (LVEF) was calculated using the following formula: LVEF (%) = (LVEDV − LVESV)/(LVEDV) × 100. Fractional shortening (FS) was calculated using the following formula: FS (%) = (LVEDD − LVESD)/(LVEDD) × 100. Diastolic Wall Strain (DWS) was used to evaluate myocardial wall stiffness and was calculated using the following formula: DWS = (LVPWs – LVPWd)/LVPWs.

### Histological analysis

The heart specimens of rats with transplanted hiPSC-CM patches were fixed with 10% buffered formalin and embedded in paraffin using a Microm STP 120 Spin Tissue Processor (STP120-3, Thermo Fisher Scientific). Serial paraffin-embedded sections (0.5 μm thick), cut using a Microm HM 430 system (MIC 990010, Thermo Fisher Scientific), were deparaffinized in xylene, dehydrated in a graded series of ethanol, and stained with H&E (Muto Pure Chemicals). The sections were then imaged using a light microscope (DM4000B; Leica). For analysis of fibrosis, paraffin-embedded sections were stained with Masson’s trichrome, and the percentage of fibrotic area in the entire tissue was measured by computerized planimetry using the MetaMorph imaging analysis software (Universal Imaging Corporation, Downingtown, PA, USA). For analysis of the transverse diameter of cardiac myocytes, paraffin-embedded sections were stained with Periodic acid-Schiff (PAS). Four to six fields from each section were randomly selected and imaged using a fluorescence microscope BZ-900 Analyzer (Keyence, Osaka, Japan). For analysis of teratomas, paraffin-embedded sections were stained with HE and imaged using the BZ-900 Analyzer.

For immunohistochemistry, paraffin-embedded sections were deparaffinized in xylene, dehydrated in a graded series of ethanol, and processed for antigen retrieval by autoclaving with 0.01 M citrate buffer. The sections were then immersed in methanol containing 3% hydrogen peroxide to inactivate endogenous peroxidase and then incubated overnight at 4 °C with anti-von Willebrand Factor (vWF; Merck Millipore, Billerica, MA**,** USA), anti-alpha smooth muscle actin (SMA; Dako, Glostrup, Denmark), anti-human nuclear antigen (Merck Millipore), and anti-cTnT (Abcam, Cambridge, UK) as primary antibodies. Subsequently, the sections were incubated with secondary antibodies, namely Alexa Fluor 488- or Alexa Fluor 555-conjugated donkey anti-rabbit or anti-mouse (Thermo Fisher Scientific) antibodies. Nuclei were counterstained with Hoechst 33342 (Dojindo). Four to eight fields from each section were randomly selected and imaged using a fluorescence microscope (BZ-900 Analyzer).

### Statistical analysis

All the results are presented as means ± SD. For the in vitro study, Student’s t-test was used for statistical analysis. For the in vivo study, comparisons of three groups were performed by one-way analysis of variance (ANOVA) followed by Tukey–Kramer post hoc analysis to evaluate statistically significant differences. Statistical significance was set at p < 0.05.

### Ethical approval and the statement of human and animal rights

Ethical approval to report this case was obtained from Osaka university hospital of ethics committee (Approval number; 14306-7). All procedures in this study involving animals were conducted in accordance with the Guide for the “Care and Use of Laboratory Animals” (National Institutes of Health publication). Experimental protocols were approved by the Ethics Review Committee for Animal Experimentation of Osaka University Graduate School of Medicine (Approval number; 30-019-020), and conducted in compliance with the ARRIVE guidelines. The Ethics Review Committee for Animal Experimentation approved animal experimental study protocols.

## Data Availability

The datasets used and/or analyzed during the current study are available from the corresponding author upon reasonable request.

## References

[1] Jeevanantham V (2012). Adult bone marrow cell therapy improves survival and induces long-term improvement in cardiac parameters: A systematic review and meta-analysis. Circulation.

[2] Salerno N (2022). Myocardial regeneration protocols towards the routine clinical scenario: An unseemly path from bench to bedside. EClinicalMedicine..

[3] Menasché P (2001). Myoblast transplantation for heart failure. Lancet.

[4] Miyagawa S (2017). Phase I clinical trial of autologous stem cell-sheet transplantation therapy for treating cardiomyopathy. J. Am. Heart Assoc..

[5] Wehman B, Kaushal S (2015). The emergence of stem cell therapy for patients with congenital heart disease. Circ. Res..

[6] da Cruz L (2018). Phase 1 clinical study of an embryonic stem cell-derived retinal pigment epithelium patch in age-related macular degeneration. Nat. Biotechnol..

[7] Menasché P (2018). Transplantation of human embryonic stem cell-derived cardiovascular progenitors for severe ischemic left ventricular dysfunction. J. Am. Coll. Cardiol..

[8] Schweitzer JS (2020). Personalized iPSC-derived dopamine progenitor cells for Parkinson’s disease. N. Engl. J. Med..

[9] Mandai M (2017). Autologous induced stem-cell-derived retinal cells for macular degeneration. N. Engl. J. Med..

[10] Stoddard-Bennett T, Reijo Pera R (2019). Treatment of Parkinson's disease through personalized medicine and induced pluripotent stem cells. Cells.

[11] Miyagawa S (2022). Case report: Transplantation of human induced pluripotent stem cell-derived cardiomyocyte patches for ischemic cardiomyopathy. Front. Cardiovasc. Med..

[12] Neofytou E, O'Brien CG, Couture LA, Wu JC (2015). Hurdles to clinical translation of human induced pluripotent stem cells. J. Clin. Invest..

[13] Ortuño-Costela MDC, Cerrada V, García-López M, Gallardo ME (2019). The challenge of bringing iPSCs to the patient. Int. J. Mol. Sci..

[14] Kawamura T (2016). Cardiomyocytes derived from MHC-homozygous induced pluripotent stem cells exhibit reduced allogeneic immunogenicity in MHC-matched non-human primates. Stem Cell. Rep..

[15] Kashiyama N (2019). MHC-mismatched allotransplantation of induced pluripotent stem cell-derived cardiomyocyte sheets to improve cardiac function in a primate ischemic cardiomyopathy model. Transplantation.

[16] Guethoff S (2013). Ten-year results of a randomized trial comparing tacrolimus versus cyclosporine a in combination with mycophenolate mofetil after heart transplantation. Transplantation.

[17] Ito E (2019). Tumorigenicity assay essential for facilitating safety studies of hiPSC-derived cardiomyocytes for clinical application. Sci. Rep..

[18] Takeda M (2018). Development of in vitro drug-induced cardiotoxicity assay by using three-dimensional cardiac tissues derived from human induced pluripotent stem cells. Tissue Eng. Part C Methods.

[19] Halbach M (2013). Electrophysiological integration and action potential properties of transplanted cardiomyocytes derived from induced pluripotent stem cells. Cardiovasc. Res..

[20] Higuchi T (2015). Functional and electrical integration of induced pluripotent stem cell-derived cardiomyocytes in a myocardial infarction rat heart. Cell Transplant..

[21] Dhahri W (2022). In vitro matured human pluripotent stem cell-derived cardiomyocytes form grafts with enhanced structure and function in injured hearts. Circulation.

[22] Kawamura M (2012). Feasibility, safety, and therapeutic efficacy of human induced pluripotent stem cell-derived cardiomyocyte sheets in a porcine ischemic cardiomyopathy model. Circulation.

[23] Mandai M (2017). Autologous induced stem-cell–derived retinal cells for macular degeneration. N. Engl. J. Med..

[24] Smagul S (2020). Biomaterials loaded with growth factors/cytokines and stem cells for cardiac tissue regeneration. Int. J. Mol. Sci..

[25] Menasché P, Vanneaux V (2016). Stem cells for the treatment of heart failure. Curr. Res. Transl. Med..

[26] Nguyen PK, Rhee JW, Wu JC (2016). Adult stem cell therapy and heart failure, 2000 to 2016: A systematic review. JAMA Cardiol..

[27] Kawamura M (2017). Enhanced therapeutic effects of human iPS cell derived-cardiomyocyte by combined cell-sheets with omental flap technique in porcine ischemic cardiomyopathy model. Sci. Rep..

[28] Shudo Y (2011). Novel regenerative therapy using cell-sheet covered with omentum flap delivers a huge number of cells in a porcine myocardial infarction model. J. Thorac. Cardiovasc. Surg..

[29] Kawamura M (2013). Enhanced survival of transplanted human induced pluripotent stem cell-derived cardiomyocytes by the combination of cell sheets with the pedicled omental flap technique in a porcine heart. Circulation.

[30] Carmeliet P, Jain RK (2011). Molecular mechanisms and clinical applications of angiogenesis. Nat. Med..

[31] Kainuma S (2015). Cell-sheet therapy with omentopexy promotes arteriogenesis and improves coronary circulation physiology in failing heart. Mol. Ther..

[32] Rademakers T, Horvath JM, van Blitterswijk CA, LaPointe VLS (2019). Oxygen and nutrient delivery in tissue engineering: Approaches to graft vascularization. J. Tissue Eng. Regen. Med..

